# Comparative evaluation of the performance of orthodontic retainers using different surface protocols: an in vitro study

**DOI:** 10.1007/s10103-025-04348-4

**Published:** 2025-02-13

**Authors:** Merve Kurnaz, Ali Arslan Nazan, Feyza Eraydın

**Affiliations:** 1 Istanbul Kent University, Istanbul, Turkey; 2 Private Company Permatter LLC, Florida, USA; 3 Gelişim Üniversitesi, Istanbul, Turkey

**Keywords:** Computer-aided design/computer-aided manufacturing, Fixed lingual retainers, Permanent retantion, Laser surface texturing, Atmospheric plasma

## Abstract

This study aims to assess the in vitro durability of Nitinol retainers, manufactured using computer-aided methods with hydrophilic or superhydrophilic surfaces to reduce debonding, alongside a commonly used composite adhesive. The 112 lower incisor teeth were embedded in blocks in pairs. Retainer wires were made up of 0.018 × 0.018 inch Nickel Titanium alloy(G4™ Nickel Titanium G&H Orthodontics, USA) by bending a robot arm. A total of 16 teeth(8 blocks) were used for each of the mentioned 7 groups Ni-Ti Retainer; Laser Textured Ni-Ti Retainer; Laser Texturing and Atmospheric Plasma Applicated Ni-Ti Retainer; Atmospheric Plasma Applicated Ni-Ti Retainer; Laser Texturing and Atmospheric Plasma Applicated Ni-Ti Retainer*2; Laser Texturing and Atmospheric Plasma Applicated Ni-Ti Retainer*3; SS-0.0018“(Morelli, Brazil). Transbond LR(3 M Unitek, California) was used. The shear bond strength tests were conducted. The Kruskal-Wallis H test was employed, pairwise comparisons followed by Dunn’s test with Bonferroni correction as a post-hoc analysis. There was no statistically significant difference between the groups for maximum force and maximum stress(p > 0.05). However, a significant difference was found in maximum elongation (p:0.0023). Pairwise comparisons highlighted significantly higher elongation values in the SS-0.0018” group. The stainless-steel wire demonstrated higher elongation values, which may offer clinical advantages in cases with higher occlusal forces and periodontal problems due to its material flexibility. Laser Texturing and Atmospheric Plasma Applied Ni-Ti Retainers exhibited higher test performance. Surface treatments applied to CAD/CAM retainers can provide an advantage by enhancing bond strength, potentially reducing the risk of debonding. These findings underline the importance of material selection and surface treatments in optimizing fixed retention strategies for long-term clinical success.

## Introduction

Orthodontic retention is defined as the prevention of teeth returning to their pretreatment positions due to insufficient bone support, accomplished through the use of removable or fixed retainers to maintain the results of orthodontic treatment [1–3]. Removable retainers can cause discomfort during speech and wear out over time, and their effectiveness relies on patient ability [4]. Fixed retainers ensure stability for anterior teeth with minimal patient compliance, and they become increasingly popular mostly in the retention of mandibular incisors, since the late 1970s [5]. Straight round/rectangular archwires were preferred which are passively bonded to the lingual surface of anterior teeth [6–8]. Zachrisson [8] proposed the use of multistranded archwires with irregular surface areas, which provide mechanical retention when bonded with composite materials.

Aycan et al. [9] roughened teeth using acid and Er: YAG or Er, Cr: YSGG laser, followed by bonding with dead wire and CAD/CAM-fabricated fiber-reinforced wires. They concluded that CAD/CAM-fabricated wires, due to their resistance to deformation, hold potential for reuse in clinical applications. The highest bond strength was observed with the combination of acid treatment and dead-soft wire. Johnson et al. [10] evaluated the bond strength of three lingual retainer wires—flat-woven (Leone), dead-soft (Ortho Classic USA), and two-stranded twisted (Leone)—using Transbond XT (3 M Unitek) and Enlight Light Cure Adhesive (Ormco). The highest shear bond strength (SBS) was observed with the flat-woven wire and Transbond XT composite.

Intraoral scanners enhance diagnosis, treatment planning, and the precision production of custom appliances, including bonded fixed retainers, through three-dimensional (3D) computer-aided design and computer-aided manufacturing (CAD/CAM) technology [10]. Memotain (CA-Digital, Mettmann, Germany), a custom-designed retainer fabricated digitally using CAD-CAM technology and made with special technology using nitinol, which provides high precision and comfort for the patient, has been introduced [11, 12]. Nitinol retainers, due to their fully adaptable and thin structure help to reduce the risk of fracture [12]. The shape-memory property of nitinol retainers allows them to return to their original shape after deformation [12]. In an in vitro study of nitinol retainers, the amount of deformation of the retainer wires was investigated, and it was concluded that no deformation was detected in the nitinol retainers [12].

Indeed, factors such as bad habits, contamination, and chewing forces can contribute to connection failures and fractures in fixed retainers [13]. The connection failures in fixed retainers can include the separation of the wire from the composite, debonding of the composite from the enamel surface, and wire breakage. The most common type of failure in fixed retainers is wire debonding from the composite [14, 15] as a separation between the adhesive and the enamel surface [16, 17].

Upon reviewing the literature, it is evident that there are studies examining leaching and cytotoxicity on retainers, as well as studies investigating their electrochemical corrosion resistance [18, 19]. Additionally, there are studies analyzing the bonding strength of retainers about the bonding agent used [20]. In this study compared the performance of Scotchbond Universal adhesive in total-etch mode with the traditional Transbond XT Primer for bonding fixed orthodontic retainers. In vitro, both adhesives showed similar bond strength and adhesive remnant index (ARI) scores. However, in the clinical phase, SB demonstrated a significantly lower failure rate over two years, suggesting it could be a reliable alternative to XT Primer for orthodontic retainer bonding.

Finlay et al. [18] investigated the corrosion products and cytotoxicity of seven fixed lingual retainers (FLRs), comparing generic and proprietary types. Metallic ions, including lead, nickel, and molybdenum, were released in varying concentrations from the wires, with some exceeding acceptable levels in drinking water. Cytotoxicity testing revealed that most wires were noncytotoxic, with slight cytotoxicity observed in only one sample at higher concentrations. The study concluded that there was no significant difference in biocompatibility between generic and proprietary FLRs, though variability in metal release was noted. A recent in vitro study compared the electrochemical corrosion resistance of six types of fixed lingual retainer wires, including 3-braided and 6-braided stainless steel, Titanium Grade 1, Titanium Grade 5, Gold, and Dead Soft wires, in Ringer solutions with pH 7 and pH 3.5 [19]. The Titanium Grade 5 wires demonstrated the highest polarization resistance and no pitting corrosion in SEM analysis, indicating superior corrosion resistance. Conversely, Gold wires showed the highest corrosion current density, and Dead Soft wires exhibited the highest corrosion rate [19]. These findings suggest that Titanium Grade 5 wires may be more suitable for clinical use due to their durability and resistance to corrosion [19].

Also, in dentistry laser texturing technology is widely employed to enhance the surface characteristics of prosthetic restorations and improve the properties of implant surfaces [21–23]. While certain surface modifications have been applied to orthodontic wires, this technology has not been utilized to increase the bonding strength of brackets or retainers. Plasma treatment, another surface modification technique, is commonly used to enhance the bonding strength of orthodontic brackets by altering their surface properties [24, 25]. However, according to the literature, these surface treatment methods have not yet been applied to retainers, which are prone to debonding.

The reviews of the literature reveal a lack of sufficient studies on nitinol retainers and surface-altering processes. Considering this, the purpose of our study was to examine the in vitro durability of a nitinol retainer that is produced using computer-aided methods and incorporates hydrophilic or superhydrophilic surfaces to reduce debonding when using the commonly encountered composite adhesive. The null hypothesis of our study posits that there is no significant difference in the tests evaluated across all groups in conjunction with the applied surface treatments.

## Materials and methods

This in vitro study investigates the bonding properties of lingual retainer wires on extracted teeth, conducted following approval from the Ethical Committee of **** University (Ref. No. 2024/86 − 63/ 06.03.2024).

According to the power analysis (G*Power software (Version 3.1.9.4, Dusseldorf, Germany)) the required sample size in each group was determined as 16 with a confidence level of 95% (1 − α), a test power of 87% (1 − β), and an effect size of *d* = 0.4 based on the study by Aycan M, et al. [9]. Therefore, the total sample size for the study was set at 112 (16 teeth x 7 group).

**Fig. 1:**
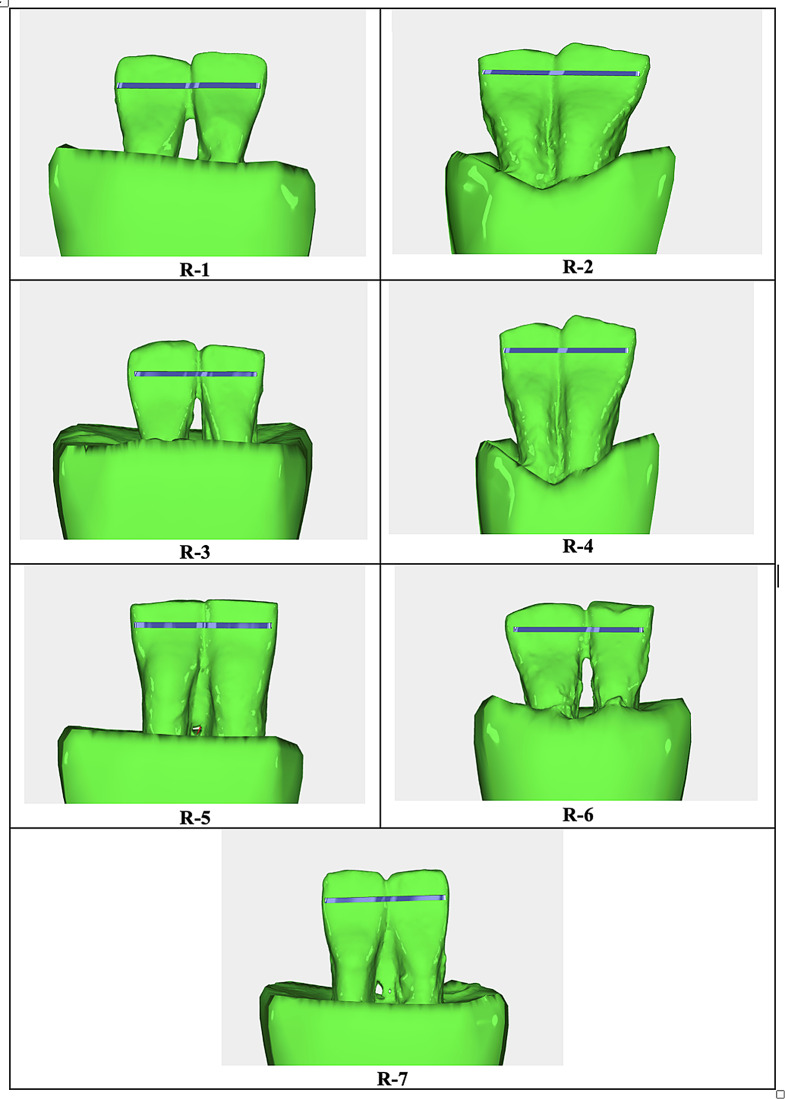
This figure shows the CAD models of fixed orthodontic retainers, displaying the detailed structure and components used in the study. R-1: 0.018 × 0.018 inch Nickel Titanium alloy (G&H Orthodontics, USA) Ni-Ti Retainer; R-2: 0.018 × 0.018 inch Nickel Titanium alloy(G&H Orthodontics, USA) Laser Textured Ni-Ti Retainer, R-3: 0.018 × 0.018 inch Nickel Titanium alloy(G&H Orthodontics, USA) Laser Texturing and Atmospheric Plasma Applicated Ni-Ti Retainer, R-4: 0.018 × 0.018 inch Nickel Titanium alloy(G&H Orthodontics, USA) Atmospheric Plasma Applicated Ni-Ti Retainer, R-5: SS − 0.0018” (Morelli, Brazil), R-6:0.018 × 0.018 inch Nickel Titanium alloy(G&H Orthodontics, USA) Laser Texturing and Atmospheric Plasma Applicated Ni-Ti Retainer*2, R-7: 0.018 × 0.018 inch Nickel Titanium alloy(G&H Orthodontics, USA). Laser Texturing + Atmospheric Plasma Applicated Ni-Ti Retainer*3

**Fig. 2:**
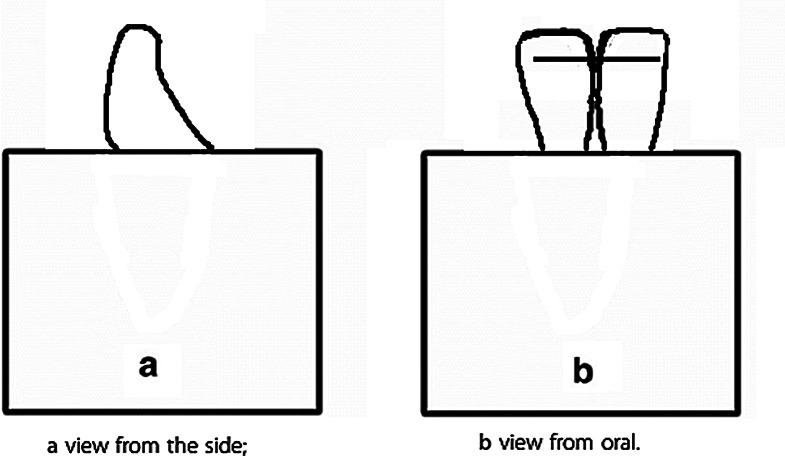
This figure illustrates the design of the testing retainer including the placement protocol used for the tests

**Fig. 3:**
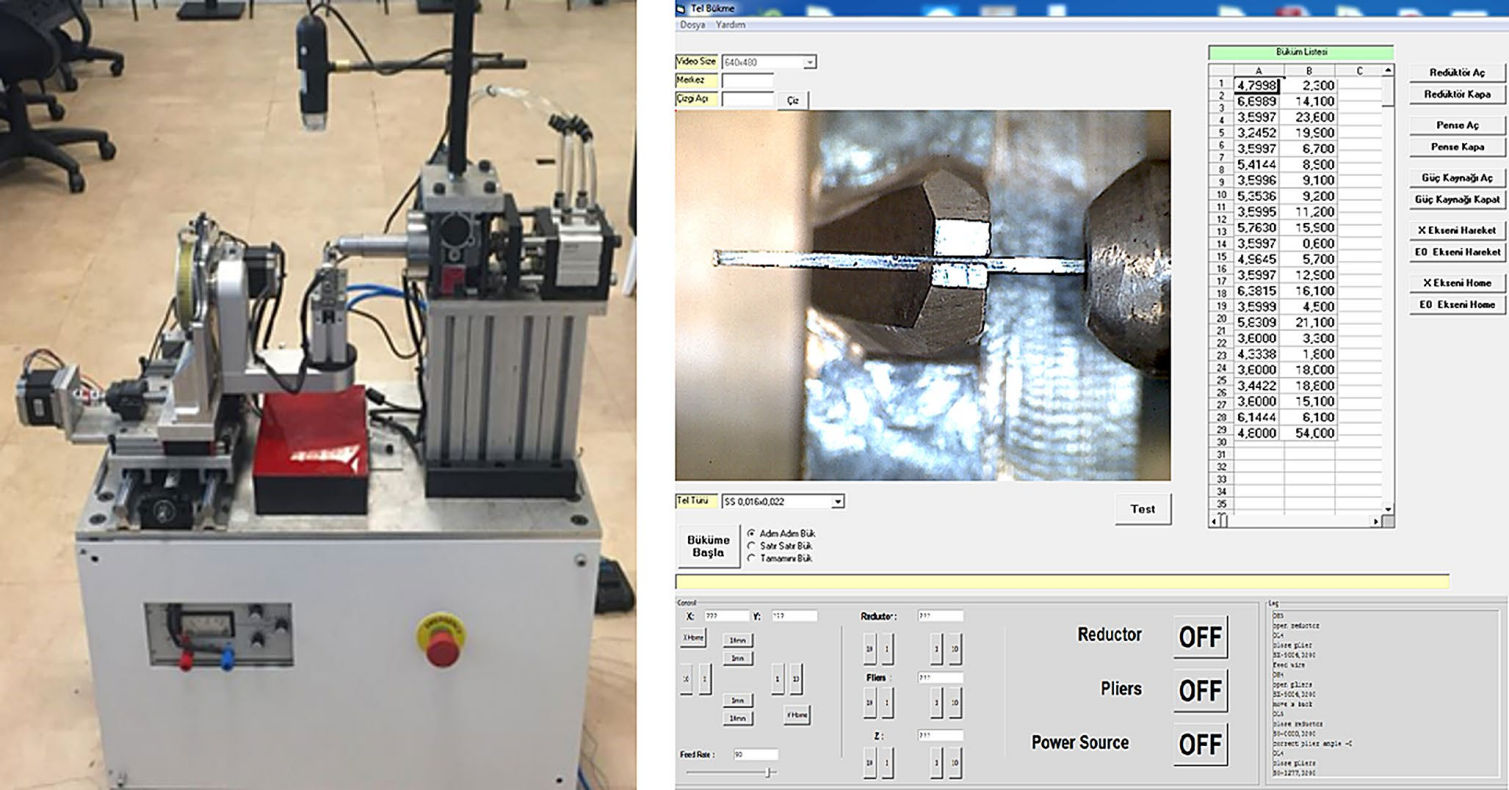
Demonstration of the process of applying bending forces to the retainers, showing the setup and how the retainers were subjected to stress in the experimental conditions

**Fig. 4:**
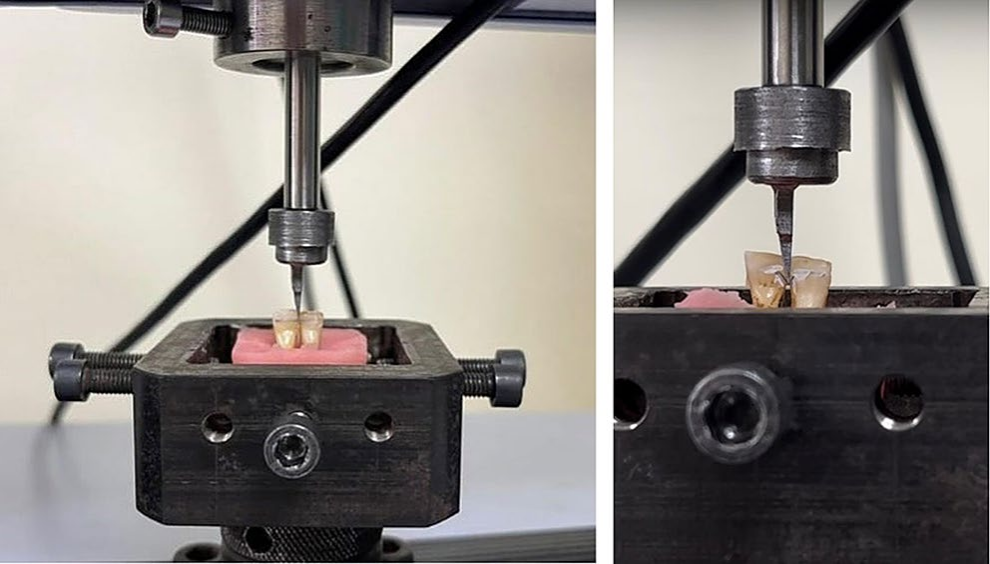
The moment of debonding during the testing procedure

The technologies and the study groups used in the production process of retainers are summarized in Table 1; Figs. 1, 2, 3 and 4. Fixed retainers were designed using Rhinoceros 3D software (Spain). They were bent by a five-axis archwire bending robot (Fig. 3). This robot was specially developed to bend nickel-titanium archwires using a heat-treatment method. Stainless-steel retainers were bent without heat treatment. A NFL-50 W (Nanosecond Fiber Laser, Han’s Laser, China) device and an Atmospheric Plasma (Tantec–PlasmaTEC-X, Denmark) device were used in order to increase bonding properties of fixed retainers. Atmospheric plasma treatment was performed using a XYZ Cartesian system-based setup, with a nozzle speed of 15 mm/min (single pass), supported by an air compressor. The laser treatment utilized a nanosecond fiber laser, operating at a scan speed of 1000 mm/s and a laser power of 50 W.

**Table 1:** Retainer groups and surface treatment properties

Groups	Material	Property
R-1	0.018 × 0.018 inch Nickel Titanium alloy (G4™ Nickel Titanium, G&H Orthodontics, USA) Ni-Ti Retainer	Only electropolishing process was applied.
R-2	0.018 × 0.018 inch Nickel Titanium alloy(G4™ Nickel Titanium, G&H Orthodontics, USA) Laser Textured Ni-Ti Retainer	Electropolishing process was applied first, followed by laser texturing.
R-3	0.018 × 0.018 inch Nickel Titanium alloy(G4™ Nickel Titanium, G&H Orthodontics, USA) Laser Texturing and Atmospheric Plasma Applicated Ni-Ti Retainer	Electropolishing process was applied first, followed by laser texturing and then atmospheric plasma treatment.
R-4	0.018 × 0.018 inch Nickel Titanium alloy(G4™ Nickel Titanium, G&H Orthodontics, USA) Atmospheric Plasma Applicated Ni-Ti Retainer	Electropolishing process was applied first, followed by atmospheric plasma treatment.
R-5	SS − 0.0018” (Morelli, Brazil)	Morelli Orthodontics (Brasil) 3-coil stainless steel material with robotic bend was used. No surface altering method applied.
R-6	0.018 × 0.018 inch Nickel Titanium alloy(G4™ Nickel Titanium, G&H Orthodontics, USA) Laser Texturing and Atmospheric Plasma Applicated Ni-Ti Retainer*2	Electropolishing process was applied first, followed by laser texturing, atmospheric plasma treatment, and then another round of electropolishing.
R-7	0.018 × 0.018 inch Nickel Titanium alloy(G4™ Nickel Titanium, G&H Orthodontics, USA) Laser Texturing + Atmospheric Plasma Applicated Ni-Ti Retainer*3	Electropolishing process was applied first, followed by laser texturingby laser texturing (with varying power and frequency), atmospheric plasma treatment, and then another round of electropolishing.

In this study, 112 lower incisor teeth were selected based on criteria such as being extracted for orthodontic or periodontal purposes; having no cavities, fillings, or fractures; and having a smooth buccal surface. The extracted teeth were primarily orthodontically indicated, typically from adolescent patients, and included mandibular incisors with orthodontic and periodontal indications from patients under 40 years of age. Teeth were stored in glass bottles containing 0.1% thymol solution and refreshed every month to ensure their effectiveness in a refrigerator at + 4 ℃ until used.

The lingual surfaces of the teeth were then cleaned using a low-speed micromotor and an oil-free polishing cup. They were rinsed with water using an air–water spray and dried. Two incisor teeth were placed side by side and embedded in acrylic blocks that were 3 × 3 cm. The blocks were then scanned using an intraoral iTero^®^ 3D scanner (Align Technology, Tempe, AZ, USA). The teeth were etched with 37% orthophosphoric acid for 20 s, rinsed for 40 s, and subsequently treated with a retainer adhesive material (Transbond LR, 3 M Unitek, CA, USA) according to the manufacturer’s instructions to fabricate the retainers. The bonding was cured from the mesial side first and then from the distal side using the VALO Ortho Cordless appliance with an irradiance of 3,200 mW/cm² (Ultradent, South Jordan, USA). Using CAD/CAM technology, the retainers in all groups were designed to have maximum contact with the tooth surfaces using the retainer material length of 10 mm. Retainer wires were made of 0.018 inch ×0.018-inch nickel-titanium alloy(G4™ Nickel Titanium -G&H Orthodontics, USA)by bending with a robot arm. A total of eight blocks were used for each of the seven mentioned groups.

0.018’’ three-loop stainless-steel robotic bended wire (Morelli, Brazil), which was chosen to evaluate a material with a similar cross-sectional area for comparison to the newly designed retainer wires.

Embedded specimens were secured in a jig attached to the base plate of the testing device. The crosshead speed was set to 1 mm/min and the maximum load necessary to debond the wire was recorded (Fig. 4). The shear bond strength tests were conducted on a Shimadzu AGS-X Universal testing machine located in **** University, Faculty of Dentistry Laboratory (Fig. 4). The cutting edge of the machine was positioned perpendicular to the interdental area of the testing application. The Maximum Force (N), Shear bond strength(SBS) (N), and Maximum Elongation were measured and recorded on a computer.

### Statistical analysis

IBM SPSS Statistics for Windows, Version 22.0 (Released 2013; IBM Corp., Armonk, New York, USA) software was used. The normality of the data was assessed using the Shapiro-Wilk test. For datasets that did not exhibit a normal distribution across three or more groups, the Kruskal-Wallis H test was employed, pairwise comparisons followed by Dunn’s test with Bonferroni correction as a post-hoc analysis. A p-value of less than 0.05 was considered statistically significant.

## Results

There was no statistically significant difference between the groups for maximum force and maximum stress (*p* > 0.05),(Table 2). However, there was a statistically significant difference between the groups for maximum elongation (*p*:0,023), (Table 2). In two-pair comparison tests (The Kruskal-Wallis H test with Bonferroni correction), it is noted that the values of elongation in the R-5 (SS-0.0018” [Morelli, Brazil]) group were significantly higher compared to the other groups. Although no statistical difference was observed, the highest values for maximum force and elongation at shear bond strength were recorded in the R-3 group [0.018 × 0.018 inch Nickel Titanium alloy(G&H Orthodontics, USA) Laser Texturing and Atmospheric Plasma Applicated Ni-Ti Retainer)], with 132.99 N and 112.95 N, respectively. The lowest values for maximum force and elongation at shear bond strength were recorded in the R-6[0.018 × 0.018 inch Nickel Titanium alloy(G&H Orthodontics, USA) Laser Texturing and Atmospheric Plasma Applicated Ni-Ti Retainer*2] group.

**Table 2:** The comparative evaluation of test results between groups

		Groups					Kruskall-Wallis H Test		
		n	Mean	Minimum	Maximum	sd	H	p	Pairwise Comparison Bonferroni correction
Maximum Force (N)	R-1	8	90,92	54,73	123,27	22,75	11,83	0,066	-
	R-2		107,20	57,32	133,48	23,07			
	R-3		132,99	62,35	208,17	44,64			
	R-4		77,92	35,41	125,42	34,42			
	R-5		84,78	26,39	114,21	31,29			
	R-6		83,90	65,60	129,97	22,88			
	R-7		95,86	71,84	128,53	18,25			
	Total	56	96,56	26,39	208,17	32,73			
Shear bond strength (N)	R-1	8	74,45	57,92	112,80	19,96	10,07	0,122	-
	R-2		109,94	87,21	132,10	22,83			
	R-3		112,95	51,04	192,88	46,18			
	R-4		71,03	51,99	111,90	27,64			
	R-5		91,46	65,11	106,42	17,03			
	R-6		75,86	54,77	121,92	26,54			
	R-7		87,89	65,59	108,42	14,45			
	Total	56	89,82	51,04	192,88	29,90			
Maximum Elongation (mm)	R-1	8	0,97	0,05	2,01	0,66		0,023*	5-1 5-2 5-3 5-4 5-6 5-7
	R-2		1,09	0,51	1,85	0,48			
	R-3		0,70	0,44	0,98	0,21			
	R-4		1,35	0,40	2,83	0,85			
	R-5		2,01	1,18	2,81	0,67	14.65		
	R-6		1,04	0,05	2,05	0,70			
	R-7		1,03	0,14	1,66	0,49			
	Total	56	1,17	0,05	2,83	0,69			

* *p* < 0,05 as statistically significant

## Discussion

Laser texturing has begun to be used in various branches of dentistry and plasma treatment is also being employed in orthodontics to enhance bonding strength [24–28]. However, based on the literature, these methods have not been previously utilized in the context of retainer applications, which are often susceptible to debonding. Current study investigated the effects of laser texturing and atmospheric plasma treatment on the retention properties of different retainer materials. Our null hypothesis stated that there would be no differences among the groups; however, it was rejected due to the observed differences in elongation values. Although no statistical difference was found, the electropolishing process, followed by laser texturing and atmospheric plasma treatments, increased both the bond strength and maximum force. The higher effects were observed when the electropolishing process was applied first, followed by laser texturing and atmospheric plasma treatment. However, adding electropolishing as the final step reduced the bond strength, while it led to an increase in elongation over time.

Memotain is a CAD/CAM-fabricated lingual retainer made from 0.014” × 0.014” rectangular nickel-titanium wire, designed to flexibly and precisely fit the patient’s lingual tooth anatomy [11, 12]. Retainers are being modified to enhance bonding and prevent fractures or dislodgement due to loss of tooth connection. The nitinol retainers used in this study fabricated using computer-aided methods and feature hydrophilic or superhydrophilic surfaces to reduce debonding. Nitinol wires also exhibit superior wettability properties, further improving their performance. Wang et al. [29] developed a simple and efficient laser-based wettability control method for titanium material using a laser texturing process, creating a surface that enables reversible wettability transition between superhydrophobicity and superhydrophilicity utilizing alternate low-temperature heat treatment and UV irradiation.

The five-strand stainless steel wires are regarded as the gold standard for bonded fixed retainers [8]. It has been reported that five-strand stainless-steel plain multistrand wire is often considered the gold standard for bonded fixed retainers due to its proven long-term success, ability to allow physiological tooth movement, and sufficient thickness to resist breakage [8]. In the current study, to evaluate the retainers with the same cross-sectional area, three-strand stainless-steel wires with a dimension of 0.018” were selected as the control group, as current wires typically measure 0.45 mm × 0.45 mm (0.018” × 0.018”). Although no significant difference was found between the groups in terms of SBS values, the control group broke at 91.46 N, whereas the 0.018” × 0.018” Nickel Titanium alloy (G&H Orthodontics, USA) laser-textured and atmospheric plasma-treated Ni-Ti retainer broke at 112.95 N. The maximum elongation was observed in the 3-coil stainless steel material with robotic bending, followed by the electropolishing process, atmospheric plasma treatment, and then nitinol. In Group 7, the lowest values were observed. Here, the surface treatments were repeated once more, indicating that the second application of the surface treatments had a negative impact. Based on these findings, clinically, the use of this wire could provide a good alternative for lifetime retention.

Aycan et al. [9] observed the highest bond strength with the combination of acid treatment and dead-soft wire. They also evaluated the bonding strength of Memotain, Er, Cr: YSGG laser treatment, and Er: YAG laser treatment on the enamel surface before bonding with the Transbond LR (3 M Unitek, Monrovia, CA, USA). From a methodological perspective, while acid is readily available in clinical settings, laser treatments are time-consuming and may not always be accessible. Modifying the surface of the retainer wire instead of the tooth could reduce chairside time and enable optimal surface treatments. In the current study, a CAD/CAM-fabricated Ni-Ti retainer was designed with laser texturing and atmospheric plasma treatment to create hydrophilic and superhydrophilic surfaces. In the current study, the laser-textured and atmospheric plasma-treated Ni-Ti retainer exhibited the highest SBS value.

Kravitz et al. [12] suggest that Memotain may be particularly useful in the upper jaw, where traditional multistranded wires often fail. The upper front teeth, with challenges such as large marginal ridges or unique shapes, complicate precise fitting with manually bent wires. Additionally, the bulky nature of retainers in the upper jaw increases the risk of breakage due to contact during chewing. Surface treatments were applied to improve bonding strength. While not statistically significant, Groups 2 and 3, with surface treatment, showed higher values. The statistical outcome is influenced by sample size, but treated samples indicate a strong affinity between the bonding material and composite. Johnson et al. [10] found the highest shear bond strength with the Leone flat-woven wire bonded to Transbond XT composite. In the current study, the highest values were observed in Group 3, where Transbond XT was used, suggesting its strong performance in future clinical applications. Additionally, a universal adhesive (Scotchbond Universal, 3 M ESPE) (SB) in total-etch mode has been found to outperform a traditional orthodontic primer (Transbond XT Primer, 3 M ESPE) (XT Primer) for bonding orthodontic fixed retainers, alongside Transbond XT Light Cure Adhesive Paste (3 M ESPE). Based on this, it can be inferred that replacing the orthodontic primer (Transbond XT, 3 M ESPE) with a universal adhesive in our study could clinically enhance performance.

A meta-analysis indicated a 35.22% failure rate for bonded retainers [30], while Koller’s study found only minor deviations between virtually planned 3D retainers and their clinical intraoral placement after bonding [31]. These findings underscore the need for CAD/CAM-supported retainers to improve bond strength and placement accuracy.

Zachrisson [32] and Oesterle et al. [33] enlarged the surfaces of both ends of 0.030’’ and 0.032’’ wires by sandblasting to enhance adhesion between metal and composite. Sandblasting resulted in increased debonding and a 217% increase in SBS for all retainer-wire combinations tested. This demonstrates that sandblasting significantly improves the clinical stability of bonded retainers by increasing micro-retention after etching [34]. This was confirmed by Reisner et al. [35], who examined the effect of enamel preparation on the SBS of orthodontic brackets across four groups: sandblasted only, sandblasted before etching, acid etched only, and buffed with a fluted bur. They concluded that sandblasting could not replace acid etching. Similarly, Cal-Neto et al. [36] found an increase in bond strength with intraoral sandblasting prior to enamel etching, though the log-rank test revealed no significant difference in clinical performance. The results of our study are consistent with the literature, suggesting that surface treatments applied to materials lead to an increase in SBS values.

Sfondrini et al. demonstrated that the mesiodistal distance between bonding points affects the mechanics of the retainer-tooth system [37, 38]. Based on these findings, it can be concluded that both the retainer’s dimensions and the mesiodistal span length influence the freedom and physiological movement of the tooth [37–39]. Bearn et al. [21] observed that a larger diameter steel wire (0.55 mm; 0.0215’’) required higher pull-out forces to debond from the tested composite compared to a thinner wire (0.45 mm; 0.0175’’). This result was likely attributed to the larger bonding surface area, rather than the wire diameters themselves. Wires with lower moduli of elasticity than regular hardened-steel wires also exhibited significantly higher debonding values [22]. Ohtonen et al. [23] found that FRC retainers positioned 1–2 mm from the incisal edge exhibited higher load values compared to those placed 4–5 mm away. Based on this finding, all groups in the current study were positioned 2 mm from the incisal edge to ensure optimal material performance. Clinically, it may be recommended to place the retainers at this distance, if possible, to achieve optimal occlusion.

To increase the clinical use of nitinol retainers, it is important to consider their disadvantages, such as high cost and longer lead times due to overseas production, which may cause delays in obtaining them for patients [11, 13].

Not using thermal cycling may seem like a limitation; however, the use of nickel-titanium material does not necessitate the incorporation of thermal cycling. In material lifetime and fatigue tests, factors such as temperature, corrosion properties, surface roughness, hardness, and cold working are key parameters [40]. However, as nickel-titanium is an intermetallic compound, it is highly sensitive to environmental temperature and exhibits complex phase changes between Austenite and Martensite at specific temperatures [40]. These complexities are directly related to phase transformation and the structural phase of the material, which is why the thermal cycling - artificial aging test in water between 5ºC and 55ºC, as defined by the ISO TR 11,450 standards, was not applied in this study. It is clear from the literature that thermal aging does not occur with the same effect factor for nickel-titanium and stainless steel materials [41, 42].

A limitation of our study is the inability to fully replicate the oral environment and periodontal tissues. Future laboratory studies are needed to accurately simulate this environment and long-term clinical follow-ups. Additionally, debonding might be influenced by patients’ biting patterns or abnormal forces from the tongue or mastication. The timesaving treatment property of nitinol retainers to help the tooth realign.

One of the strengths of our study is that, to the best of our knowledge, it is the first to perform retainer bonding through surface treatment. The achievement of higher values suggests that this method holds promising potential. Clinically, modifying the wire rather than the tooth surface could provide a more easily applicable alternative.

Alternatively, specialized methods such as heat treatment and electropolishing can directly impact the material strength and elongation percentage. Based on these results, a new study could be conducted to improve retention durability.

## Conclusion

The stainless-steel wire demonstrated higher elongation values, which may offer clinical advantages in cases with higher occlusal forces and periodontal problems due to its material flexibility. While no statistical differences were observed among the groups, the Laser Texturing and Atmospheric Plasma Applied Ni-Ti Retainer group exhibited higher test values, while the group subjected to two surface treatments showed the lowest values. Clinically, in cases with a higher risk of occlusal forces, stainless-steel wire may be preferred, as its material elongation can become an advantage. Surface treatments applied to CAD/CAM retainers will provide an advantage in increasing bond strength.

## Data Availability

The data is protected on an encrypted computer and can be accessed when needed.
